# Does greenspace influence the associations between ambient temperature and violent crime? An observational study

**DOI:** 10.1088/1748-9326/adef6a

**Published:** 2025-07-24

**Authors:** Seulkee Heo, Hayon Michelle Choi, Scott W Delaney, Peter James, Michelle L Bell

**Affiliations:** 1 School of the Environment, Yale University, 195 Prospect St, New Haven, CT 06511, United States of America; 2 Harvard T.H. Chan School of Public Health, Harvard University, 677 Huntington Ave, Boston, MA 02115, United States of America

**Keywords:** crime, violence, temperature, greenspace, recreational park

## Abstract

Despite the growing evidence on the associations between greenspace and violent crime, there is a lack of research on the urban greenspace’s influence on the associations between ambient temperature and violent crime. This observational study examined the risk differences by community’s greenspace level using various greenspace indicators. Our time-series analysis modeled the associations between daily mean temperature (°C) over two lag days (lag0–1) and daily counts of violent crime during summer (May–September) in each ZIP code in Chicago, IL (2001–2023), adjusting for confounding factors. Our random-effects meta analysis analyzed estimated the pooled relative risk (RR) at the 80th summer temperature percentile compared to the reference temperature (10th percentile) across the ZIP codes. Our meta-regressions analyzed how the ZIP code-specific relative risks (RRs) differ by the number of parks, sum of park areas, percentage of vegetated area, percentage of recreational vegetated area, vegetation density (30 m), percent tree coverage, and percent street-level tree coverage aggregated at the ZIP code level. A total of 1075 959 counts of violent crime were included in our analysis. We found 8% (95% CI: 7%–10%) higher risk of violent crime incidents when the daily mean temperature was at the 80th percentile (25.9 °C) compared to the reference temperature (8.6 °C). The pooled RR was significantly lower in ZIP codes with the highest vegetation density (RR = 1.085 [95% CI: 1.040–1.131]) compared to those with the lowest vegetated density (RR = 1.124 [1.088–1.162]). The RR was significantly lower in ZIP codes with the highest percentage of tree coverage (RR = 1.088 [1.046–1.132]) compared to the ZIP codes with the lowest percentage of tree coverage (RR = 1.123 [1.086–1.162]). The observed results indicate that greenspace can be beneficial in reducing the associations between heat and violent crime. The results should be considered in urban greenery planning and policies to reduce violent crime.

## 1. Introduction

Numerous studies have suggested that violent crime rises with higher temperatures [1]. The temperature-violence relationships across different regions of the United States have been well-documented in literature. For instance, a study in Philadelphia, PA, 2012–2015 showed that the violent crime rate was 9% higher when the mean daily Heat Index reached the 99th percentile compared to the median [2]. The advanced statistical modeling in recent studies benefited from exploring potential non-linear exposure-response associations moving beyond traditional linear associations [3, 4]. The observed exposure-response relationship curves align with well-established theories on the biological mechanisms suggesting higher aggressions at temperature extremes (e.g. very hot or very cold) due to discomfort [5]. Otherwise, the negative affect escape theory suggests that aggression levels can increase at moderately high temperature due to discomfort, but extremely high temperature can reduce aggression and prioritize escape behaviors (e.g. leaving the current location, hydrating) [5, 6]. However, the spatial scales of previous studies largely remain at relatively coarse levels (e.g. cities, counties). The lack of high-resolution spatial analysis of heat-crime associations limits understanding of intra-city variations and the disparities in vulnerability to temperature’s impact on violent crime in different neighborhoods. High-resolution analysis of smaller urban areas can explore how neighborhood characteristics modify the associations between temperature and violent crime, highlighting risk disparities.

Greenspace offers well-established social and health benefits, helping mitigate the environmental challenges of rapid urbanization, fostering social connections, and promoting physical and psychological health [7]. Although definitions vary across disciplines, greenspace generally refers to areas covered with vegetation such as forests, agricultural fields, urban parks, recreational open spaces, green roofs, gardens, or tree canopies. Greenspace in urban settlements is often referred to as urban greenspace [8]. A growing literature suggests that greenspace can be beneficial in lowering crime rates [4, 9, 10], a public health concern adversely impacting well-being and quality of life [11]. Recent studies articulated plausible mechanisms including social cohesion, civic pride, physical activities, mental health improvement, perception of order, and ambient heat reduction [10, 12]. In contrast, some studies suggested increased crime or violence related to greenspace. Dense and low-lying trees can facilitate criminal activities by obstructing visibility and providing cover [13]. Some studies suggested fear of crime in greenspace such as parks and green areas in neighborhoods especially among women [14–16]. Positive associations between vegetation and crime observed in studies underline the need for careful planning of urban greenspace and the complexities of the relationship between greenspace and health [17]. Also, the inconclusive findings on the relationship between greenspace and violent crime underscore the need for further research, particularly to better understand how the characteristics of greenspace (e.g. greenspace type) influence these associations [18].

Despite greenspace’s heat mitigation and psychological effects [19], there is limited research on the role of urban greenspace in modifying the relationship between ambient temperature and violent crime. Furthermore, greenspace as a potential modifier for environmental exposure and outcomes (e.g. health outcomes) has been mostly measured by vegetation density presented by the satellite image-based gridded data [9]. There are various types of greenspace metrics to measure particular aspects of greenspace. Sum of park area or percent land covered by recreational vegetation represent the density of urban park areas. The percent land covered by trees is particularly relevant for the amount of tree canopy, which has a stronger temperature cooling effect [20]. Considering the diverse greenspaces characteristics, examining various greenspace aspects in relation to modifying heat-crime associations is essential to support decision-making for urban greenery strategies.

This study utilized high-resolution crime statistics and environmental data to investigate the relationship between ambient temperature and violent crime, and how this relationship varies with neighborhood greenspace. We utilized various metrics of greenspace to assess the potential role of greenspace in modifying the associations between temperature and violent crime. We specifically focused on the recreational functions of greenspace and vegetation types (e.g. tree canopy vs. non-tree vegetation). This study will offer valuable insights into the overall impact of greenspace on the relationship between temperature and violent crime and how this influence varies based on indicators representing specific greenspace aspects.

## 2. Method

### 2.1. Study population

This time-series analysis focused on all ZIP codes (*n* = 61) in Chicago, IL, the third largest city in the US with an approximate 2746 388 population in 2020 [21]. Chicago’s spatial variation in crime has historically made it a key site for studying neighborhood effects on crime examining how specific locations influence criminal behavior within neighborhoods [22]. Our study period was 2001–2023.

We focused on summer warm months (May–September), which is effective in examining the impact of short-term exposure to heat stress without influences of confounding factors in colder months (e.g. changes in behaviors or weather). As individuals acclimate to seasonal temperature norms transitioning from colder to warmer months [23], season-specific analysis can help assess the impact of abnormally high temperatures on violence in acclimated populations.

### 2.2. Outcome

Crime data (2001–2023) were obtained from the public data source of the Chicago Police Department’s Citizen Law Enforcement Analysis and Reporting system [24]. The data provided information on the date of the incident, code for crime types (Illinois Uniform Crime Reporting code), and coordinates of the incident location. Our definition of violent crime included codes for homicide, assault, battery, domestic violence, intimidation, and aggravated ritualism (table S1). We calculated the daily sum of violent crime for each ZIP code.

### 2.3. Exposure data

We obtained the 4 km gridded modeled data for daily temperature and dew point temperature data, 2001–2023 from the parameter-elevation regressions on independent slopes model (PRISM) [25]. This modeling data combine factors including atmospheric conditions, elevation, and terrain to generate gridded estimates for meteorological variables across the US. We estimated ZIP code-specific daily mean temperature and dew point temperature by calculating the area-weighted average of pixel values within each ZIP code. The calculated daily mean temperature and dew point temperature were linked to the daily sum of violent crime counts based on the matching ZIP code. In assessing the impact of temperature on violent crime, our model included dew point temperature as a confounder to account for human physiological responses to moisture levels and distinguish dew point temperature’s impact on the outcome.

We calculated multiple greenspace-related metrics, focusing on three primary approaches: (1) the abundance of healthy vegetation, including trees and grass; (2) the physical extent of land covered by green land use or recreational greenspace (e.g. parks); and (3) the presence of vegetation within street scenes. The first approach is based on the temporal remote-sensing data and quantifies the greenness and health of vegetation within a region. The second approach evaluates greenspace provision within regions by calculating the percentage of land covered by vegetation or designated for green land use, relative to the total area. The final approach emphasizes the visual impact of greenness, utilizing street view images to evaluate the presence of trees and grasses along streets where daily activities frequently occur.

Our first greenspace metric was the enhanced vegetation index (EVI) derived from Landsat Collection 2 Tier 1 Level 2 images (30 m grid resolution), collected every 8 d. EVI represents vegetation density and healthiness (e.g. greenness) for gridded areas. This index is an improved version of the normalized difference vegetation index calculated by the vegetations’ reflectance values of near-infrared light and red light. EVI adjusts for vegetation saturation, soil background effects, and atmospheric interference. EVI ranges from −1 to 1, with higher values indicating larger amount of vegetation. We calculated aggregated EVI values for each ZIP code and compared the averages between 2001–2011 and 2012–2023, to assess temporal greenspace changes. The results (figure S1) showed minor differences in EVI between the two periods, with an average increase of 0.037 and a maximum of 0.100.

We calculated the percentage of vegetated land for each ZIP code using 30 m gridded rasters from the National Land Cover Database (NLCD) [26], which is nationally consistent reference for land cover classification in the US. The NLCD classifies green land cover types (e.g. forests, grasslands) approximately semi-annually. We calculated the area-weighted pixel values using the obtained datasets between 2001–2021 and averaged the yearly values for each ZIP code area.

We used the 2023 OpenStreetMap polygons dataset [27] to derive other greenspace metrics. The high accuracy of OpenStreetMap-based greenspace classifications for Chicago, IL has been validated in previous research [28, 29]. We evaluated the completeness [30] of OpenStreetMap data against the NLCD. We found a completeness of 71.5% for greenspace features of the OpenStreetMap for the study area, suggesting a good agreement between the OpenStreetMap and the NLCD and a reasonable representation of the OpenStreetMap. Based on this, we incorporated the OpenStreetMap data into our analysis as a reliable dataset for identifying recreational greenspace. We calculated the percentage of recreational vegetation land for the ZIP codes focusing on parks, cemeteries, open greenspace, forests, and national parks. Next, we calculated the number of parks using the polygons of these greenspace types for each ZIP code. The sum of park area (km^2^) was calculated for each ZIP code. The greenspace, derived from the NLCD for 2011 and 2021 [26], showed negligible changes over time during the study period (figure S2). Given this minimal change, we assumed that the OpenStreetMap could be representative of the greenspace conditions throughout the study period despite their cross-sectional 2023 measurement.

Regarding the vegetation type (e.g. tree vs. grass), we calculated the proportion of area covered by tree canopies using the MOD44B dataset from NASA MODIS’s Vegetation Continuous Fields product, which offers global tree cover maps at a 250-meter resolution per pixel. The annual MOD44B rasters of tree cover fractions during 2000–2020 were overlaid, and the average tree cover was calculated across all years at the grid level. Then, we calculated area-weighted ZIP code-specific averages based on the grid values overlapping with areas and boundaries of each ZIP code.

We used the percent street-view trees, which typically refers to trees visible along streets observed in street-level imagery. This metric, estimated via Google Street View images and deep learning, represents how much of the visible ground-level scene is occupied by vegetation and is described elsewhere [31]. In brief, the percent coverage scores for vegetations were measured as percent pixels of trees and vegetation other than trees in a Google Street View image. These percent coverage scores were averaged to a 100 m raster for the contiguous US. ZIP code means were calculated by averaging raster pixel values per ZIP code over the study period. The street-view tree metric dynamically measures greenspace in activity spaces (e.g. pedestrian ways) whereas percent tree coverage and park area sums are area-based metrics at the ZIP code level [32].

### 2.4. Covariates

We used ZIP code-level socioeconomic status (SES) variables as covariates. Adjusting for SES, a common determinant of community well-being and environment, can offer more reliable estimates of how greenspace modifies the temperature-violent crime associations. We used ZIP code-level SES variables for each year between 2011–2022, obtained from the American Community Survey [33], similar to those employed in previous studies [34]. We calculated population-weighted averages of each SES variable through 2011–2022 for ZIP code-level median household income, percent of population in poverty, percent of population age ⩾65 years, and age-standardized percent of population with ⩽high school education. Then, these averages of each SES variable in addition to ZIP code-level population size were merged with the study ZIP codes. We considered the proportion of population age ⩾65 years as a potential effect modifier, as participating in criminal activities tends to decline with age [35]. We obtained the 2022 Area Deprivation Index, a comprehensive indicator combining factors of income, education, employment, and housing quality of the American Community Survey at the Census Block Group level. Deprivation can be correlated with greenspace and public well-being [36]. This index ranges from 1–100, representing the percentile ranking of deprivation levels (1 = the least disadvantaged area, 100 = the most disadvantaged area). Each ZIP code was assigned the Deprivation Index value corresponding to the Census Block Group within which it is located. In effect modification analysis, we assessed how temperature-violent crime associations varied by SES. We adjusted for multiple individual indicators to evaluate their distinct impacts. We also used the Deprivation Index only to reduce redundancy and multicollinearity while capturing the multidimensional nature of community SES.

### 2.5. Statistical analysis

We applied two-stage analyses, commonly used in time-series analysis for daily data [37]. In the first stage, we applied a generalized additive model (GAM) to derive ZIP code-specific estimates for the exposure-response associations between the lagged effects of temperature, averaged by daily mean temperature on the incident day and a prior day (i.e. lag0–1), and daily count of violent crimes. GAM enables non-linear exposure-response relationships and facilitates the identification of inflection points, such as threshold temperatures [38]. Non-linear functions are usually centered on a reference value, enabling the interpretation of effects relative to it without affecting model fit [39]. We used the 10th percentile of temperature values in warm months as the reference temperature, consistent with previous research to facilitate result comparison [2, 40]. We estimated relative risks (RRs) and respective 95% confidence intervals (CIs) at the 80th percentile temperature (close to the peak association observed for the data) compared to the 10th percentile temperature. The models adjusted for the long-term trend, daily dew point temperature, and the day of the week (appendix).

In the second stage, we used a random-effects meta analysis with the restricted maximum likelihood estimator to estimate a city-average exposure-response relationship [37]. This meta analysis pooled the ZIP code-specific estimates from the first-stage model.

We examined the effect modification on the associations between temperature and violent crime for the study ZIP codes, using a random-effects meta-regression model. This meta-regression assessed the heat-violent crime association size (i.e. RR of violent crime at the 80th vs. 10th temperature percentile) separately for each level of effect modifiers (i.e. moderators). The coefficients of effect modifiers indicate how the outcome variable (i.e. effect size of the temperature-crime associations) changes with respect to the level of effect modifier. We grouped every effect modifier (e.g. EVI) into tertile groups (i.e. T1–T3; T3 denotes the highest level). Then, we conducted an individual meta-regression for each effect modifier, and the association sizes were estimated for each tertile group of the given effect modifier. The significance of effect modification by each indicator was evaluated using the *p*-value from the omnibus test, which explains a significant proportion of the variability in effect sizes. As a post-hoc test, we performed a random-effects multivariate meta-regressions separately for each greenspace metric identified as a significant effect modifier in the prior single meta-regression. These models assessed the heat-violent crime association size (i.e. RR) separately for each level of an effect modifier. Next, we calculated the differences in log RR for the 2nd (T2) and 3rd (T3) tertile groups compared to the reference 1st tertile group (T1) and reported the corresponding *p*-values, indicating the significance of the effect size difference between the groups. In these models, we adjusted for significant SES-related effect modifiers (e.g. based on the prior single meta-regressions) to control for potential confounding of ZIP code-specific SES on the effect modification by greenspace. The socioeconomic factors that were not significant from the single-variable meta-regressions were not adjusted.

## 3. Results

Our analysis included 1075 959 counts of violent crime. The descriptive statistics (table 1) showed 5.3 for the ZIP code-level average count of violent crimes per day. The daily mean temperature in warm months ranged 2.5 °C–33.3 °C with an average of 20.8 °C. The number of parks ranged from 3–98 among the study ZIP codes. The average percentage of vegetated land across the study ZIP codes was 4.1%, while the average percent tree coverage was 9.4%.

**Table 1.** Descriptive statistics of the ZIP code-level temperature and crime data.

Variable	Mean	SD	Minimum	Q1	Median	Q3	Maximum
Area of ZIP codes (km^2^)	9.2	7.9	0.3	4.5	9.2	12.2	42.1
Violent crime count/day (n)	5.3	5.5	0.0	1.0	3.0	8.0	41.0
Daily temperature (°C)	20.8	5.0	2.5	17.7	21.6	24.5	33.3
Daily dew point temperature (°C)	13.9	5.2	−4.7	10.8	14.5	17.9	26.0
*Greenspace*							
EVI	0.154	0.050	0.024	0.127	0.158	0.184	0.252
Number of parks	33.1	20.1	3.0	19.0	27.0	49.0	98.0
Sum of park area (km^2^)	1.5	1.9	0.0	0.3	0.6	2.3	6.4
Percentage of vegetated land (%)	4.1	5.5	0.0	0.7	1.5	5.6	24.1
Percentage of recreational vegetated land (%)	12.2	11.6	0.2	3.6	8.2	19.2	46.3
Percent tree coverage (%)	9.4	2.9	3.8	7.3	9.9	11.6	16.9
Percent coverage of street-view tree (%)	17.7	7.5	1.2	12.6	19.1	22.6	32.3
Percent coverage of street-view vegetation other than tree (%)	5.1	2.5	0.5	3.1	5.6	7.2	9.1
*Socioeconomic status*							
Population size	47 424	26 005	604	28 795	46 137	67 623	111 969
Percentage population with ⩽high school education (%)	36.1	19.1	4.2	23.6	37.8	48.2	70.7
Percentage of population in poverty (%)	14.4	10.2	0.0	5.9	12.0	14.4	40.5
Percentage of population age 65+ (%)	13.6	4.2	3.0	10.8	13.6	16.6	23.0
Median household income (USD)	63 873	29 110	21 370	40 912	59 484	87 076	129 475
Deprivation Index	4.1	2.2	0.0	2.0	4.0	6.0	9.0

Figure S3 shows the map of crime, temperature, and greenspace data for each ZIP code. The map displaying the number of parks revealed slightly different geographic patterns compared to the maps of other greenspace metrics. EVI showed similar patterns with street-view trees or vegetation other than trees.

The ZIP code-specific mean temperature during 2001–2023 was negatively correlated with percent vegetated land (*r* = −0.12), percent recreational vegetated land (*r* = −0.39), sum of park area (*r* = −0.57), and percent tree coverage (*r* = −0.36) (figure S4). It had a moderately high positive correlation with deprivation level (*r* = 0.44) (figure S4).

**Figure 1.**
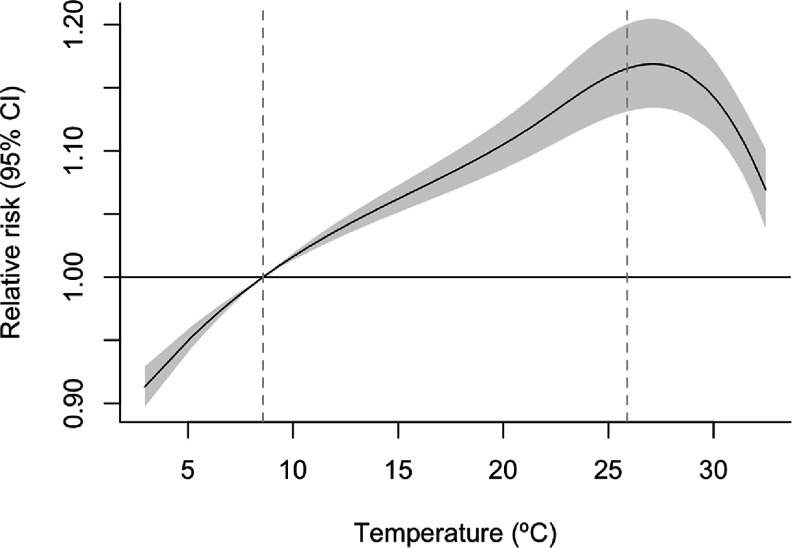
Pooled exposure-response relationship between violent crime and daily mean temperature (lag0–1) in warm months (May–September) in Chicago, IL (2001–2023).

The associations between temperature and violent crime counts pooled across the ZIP codes are shown in figure 1. The risk of violent crime steadily increased from 2.5 °C to a peak approximately at 27 °C. After this peak, the curve exhibited a steep decline in risk, resulting in an inverted J-shaped relationship. The pooled RR of violent crime at the 80th temperature percentile (25.9 °C) compared to the 10th temperature percentile (8.6 °C) as a reference temperature was 1.085 (95% CI: 1.070–1.100), indicating an 8% higher violent crime rate when the daily mean temperature reached the 80th percentile compared to the 10th percentile (*I^2^* = 29.8%).

The first dashed line represents the 10th percentile temperature, while the second dashed line marks the 80th percentile temperature.

The effect modification of each ZIP code-level moderator, based on the simple meta-regression is shown in figure S5. EVI was a significant effect modifier to lower associations between temperature and violent crime (*p*-value = 0.077) at a significance level of 0.1. ZIP codes in the highest tertile of vegetation measured by EVI (T3) exhibited a significantly lower RR compared to the other two tertile groups with less vegetation. The percentage of vegetated land was a significant effect modifier for the heat-crime association (*p*-value = 0.020). The RRs significantly differed by the percentage covered land by tree canopy (*p*-value = 0.061), with the lowest RR for the ZIP codes grouped into the highest tree canopy coverage tertile group (T3). The number of parks, the sum of park area, and the percentage of land covered by recreational vegetation were not a significant effect modifier.

**Figure 2.**
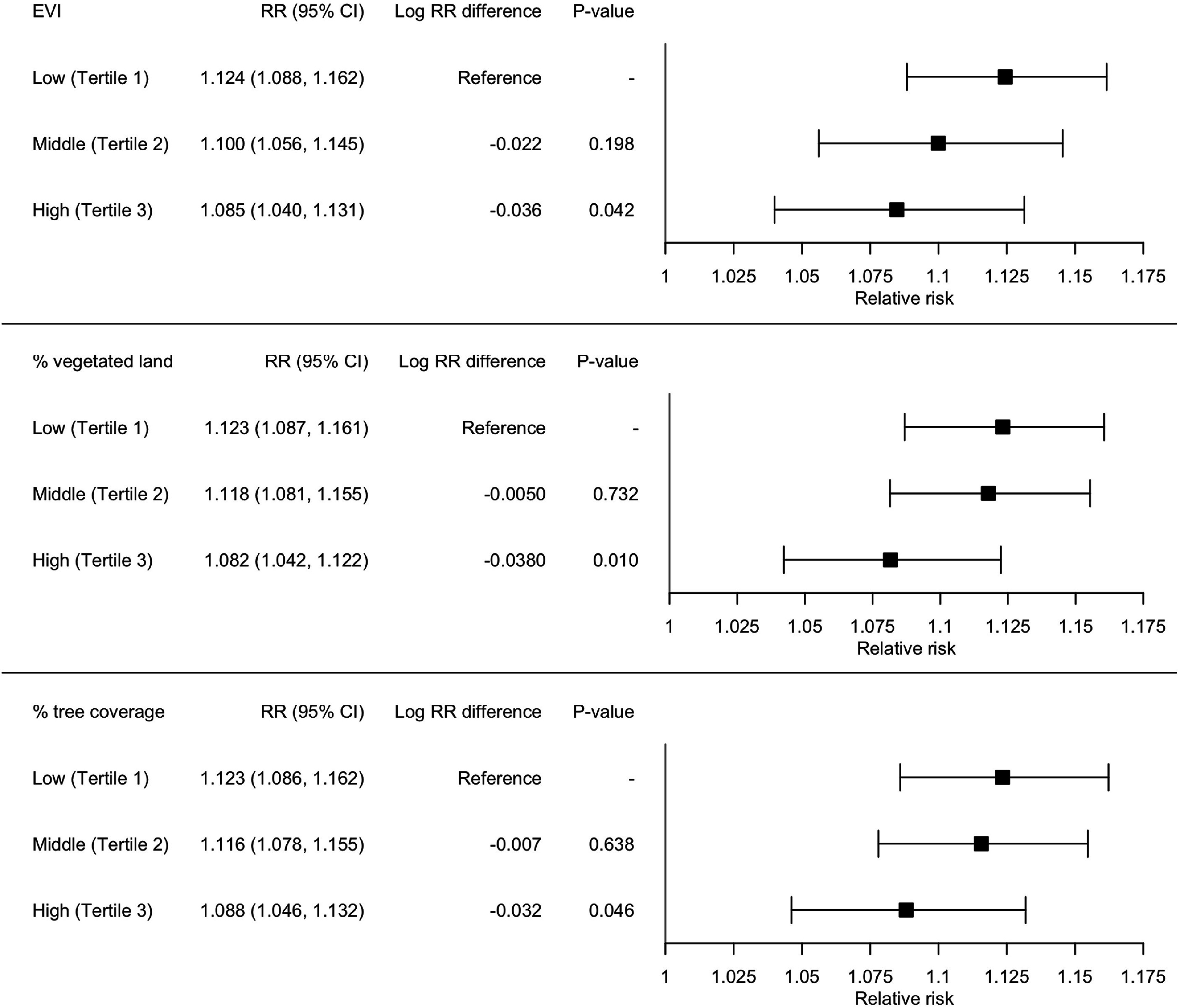
Effect modifications by greenspace on the relationship between temperature and violent crime counts. The results were adjusted for the community-level deprivation level. *P*-values are for the coefficients of each tertile group compared to its reference group (i.e. Tertile 1).

According to the multivariable meta-regressions adjusted for ZIP code-level deprivation level (figure 2), the RR in the highest EVI group (Tertile 3) had a significantly lower RR (at the 80th temperature percentile compared to the 10th temperature percentile; 1.085, 95% CI: 1.040–1.131) than the lowest EVI group (Tertile 1) for which RR = 1.124 (95% CI: 1.088–1.162). The RR in the third tertile group (RR = 1.057 [95% CI: 1.033–1.081]) had a significantly lower association than the first tertile group for percent vegetated land (RR = 1.100 [95% CI: 1.076–1.126]) (*p-*value = 0.050). The RR for the third tertile of percent tree coverage (RR = 1.088, 95% CI: 1.046–1.132) had significantly lower RR than the first tertile group of percent tree coverage (RR = 1.123, 95% CI: 1.086–1.162) (*p*-value = 0.046). Among the SES moderators, the Deprivation Index was a significant effect modifier (*p*-value = 0.002) to explain the differences in RR across the tertile groups (figure S5). Hence, in these post-hoc analysis of effect modifications, we adjusted the categories of the Deprivation Index in assessing the effect modifications by greenspace metrics.

## 4. Discussions

Our findings revealed significant increases in violent crimes associated with rising temperatures during warm months, illustrated by an inverted J-shaped exposure-response curve. Our results for temperature-violent crime associations are comparable with some previous research. A multi-county study in the US found an inverted U-shaped response curve and 11.9% (95% CI: 11.6, 12.3) higher violent crime risk for each 10 °C increase in daily temperature [1]. Another study observed a J-shaped relationship between temperature in all seasons (2011–2021) and crime counts in Chicago [41]. Hou *et al* also found that the lagged effects of temperature on crime counts persisted on lag0–1 d but became insignificant from lag day two, indicating a short-term impact of temperature on crime. The differences in exposure-response relationship curves among studies can be attributed to variations in statistical modeling (e.g. length of lag), season-specific analyses, study periods, definitions of crimes, and the level of crime count aggregation (e.g. city vs. ZIP code). Overall, these studies, including ours, suggest that elevated temperatures can have short-term effects on increasing violent crime risks.

Research has shown variations in violent crime rates based on factors including population density and racial demographics [32, 42]. It is important to adjust for these contextual factors when assessing the influence of greenspace on the temperature-crime associations. We found that ZIP codes with higher EVI (i.e. vegetation), percentages of vegetated land, and/or tree coverage exhibited weaker associations between summer temperatures and violent crime after adjusting for ZIP code-level deprivation. It is also crucial to consider specific attributes of greenspace (e.g. vegetation type); however, significant research gaps persist. Our findings of significant effect modification by the percentage of tree coverage, reflecting reduced associations between violent crime and temperature, highlight heat mitigation as a plausible pathway. This is particularly supported by evidence showing greater air-cooling effects from tall vegetation (e.g. trees) with large canopy, compared to low-height vegetation (e.g. grass) [43]. However, no effects were found for street-view trees or other street-view vegetation potentially due to the complex and context-specific impact of street trees. Street trees can lower heat exposure on pedestrian roads [44, 45], but this effect may be specific to roads only and not notable in non-road locations [46]. Also, street trees can trap heat within road canyons during the day and release it at night, contributing to elevated indoor temperatures [47, 48]. Further research is needed to determine whether this result can be generalized to other geographical regions.

Besides the air-cooling effects of greenspace, restoration effects may be associated with reduced impacts of heat on violent crimes. Viewing vegetation can immediately evoke positive emotions and reduce stress [49]. Greenspace also fosters social cohesion (e.g. a sense of safety and mutual respect), enhancing well-being and mental health [49]. It is assumed that populations in greener neighborhoods might have more resilience to the heat-aggression associations.

We did not identify significant risk differences based on recreational greenspace (e.g. parks, recreational vegetated lands). These metrics primarily capture the functions of managed vegetation for various activities (e.g. social, physical, recreational). Factors such as the quality, safety, and capacity of managed parks can significantly influence their use and visitation [50]. We lacked information on amenities, safety, or facilities within parks, which are critical quality determinants. Therefore, we could not fully assess how these park aspects might have influenced the observed effect modifications, warranting future studies.

Our study has strengths. To our knowledge, this is the first to examine effect modifications by greenspace on associations between temperature and violent crime in Chicago, IL. We investigated various greenspace metrics, previously studied for the impact of temperature on human health outcomes but not for violence. Second, the spatial unit of our analysis was at a high resolution (i.e. ZIP codes). The current evidence on temperature and crime is limited to coarse spatial scales of analysis (e.g. city, county), hindering examinations of the intra-urban spatial heterogeneity and risk disparities of more vulnerable neighborhoods due to contextual and environmental characteristics [2, 51]. Furthermore, our time-series modeling approach provided a flexible assessment of exposure-response relationships between temperature and crime over the long study period across 23 years, adjusting for long-term trends.

Our study has limitations. First, it is well known that police reporting data have limitations. The original data may contain inaccuracies or omissions due to human error, and we were unable to address these potential issues, as the data rely solely on reported incidents. While incident reports have limitations, thorough analysis of this data can provide valuable insights into temporal and spatial crime patterns [22]. Although we carefully investigated potential unreliable fluctuations or outliers in the daily violent crime trends for each ZIP code, some uncertainty in the reported crime levels may persist. Future research that validates the reported crime data against original incident reports could be valuable. Second, we did not explore or validate well-known theories such as the ‘heat-aggression theory’ or ‘the routine activity theory’ due to the lack of information on aggression levels of individuals or human mobility. Additionally, our models did not quantify or compare the specific pathways (e.g. heat mitigation, restoration) through which greenspace may modify the impact of temperature. Third, we did not have information on different tree species although our study distinguished vegetation types such as trees and grass, which have different air-cooling effects [52]. Also, the EVI estimates may not indicate physical characteristics (e.g. tree height, tree crown volume) of urban greenspace [53]. Cooling effects vary depending on tree species, height of trees, and amount of leaves [54]. Identifying the most effective tree species for reducing heat stress and violent crime risk lies beyond the scope of this study and warrants further investigation in future research. Fourth, due to the absence of longitudinal data, we relied on cross-sectional data for greenspace (2023) and SES indicators (2011–2022) as effect modifiers, rather than using statistics representing the entire 23 year study period. This misalignment between analysis periods of exposure-response functions and effect modifiers may bias effect modification estimates or introduce uncertainty about temporal changes. Nonetheless, employing area-specific representative statistics in meta-regressions of area-specific coefficients is a widely accepted approach.

## 5. Conclusion

We found that days with higher temperatures during warm months had higher risks of violent crime incidents than days with lower temperatures. This positive association between temperature and violent crime was modified by greenspace metrics, such as satellite-based vegetation density, percentage of all vegetated areas, and the percentage of tree coverage for the study ZIP codes. The risk of violent crime in more vegetated areas can be plausibly explained by the positive effects of greenery on human well-being and the cooling impact of tall and denser vegetation, such as trees, which has greater cooling effects than other types of vegetations. This study provides valuable insights to guide urban planning and inform policies aimed at mitigating temperature-related crime. Future research is needed to explore the mechanisms underlying effect modifications, which may vary based on different greenspace quality and tree species.

## Data Availability

The data are openly avilable from the PRISM Climate Group at [https://prism.oregonstate.edu], the Chicago Data Portal at [https://data.cityofchicago.org/Public-Safety/Crimes-2001-to-Present/ijzp-q8t2/about_data], OpenStreetMap at [https://download.geofabrik.de/north-america/us.html], and US Census Bureau at [https://data.census.gov/table].

## References

[1] Berman J D, Bayham J, Burkhardt J (2020). Hot under the collar: a 14-year association between temperature and violent behavior across 436 U.S. counties. Environ. Res..

[2] Schinasi L H, Hamra G B (2017). A time series analysis of associations between daily temperature and crime events in Philadelphia, Pennsylvania. J. Urban Health.

[3] Baryshnikova N, Davidson S, Wesselbaum D (2022). Do you feel the heat around the corner? The effect of weather on crime. Empiri. Econ..

[4] Heo S, Choi H M, Berman J D, Bell M L (2025). Temperature, violent crime, climate change, and vulnerability factors in 44 United States cities. Environ. Int..

[5] Cohn E G, Rotton J (2000). Weather, seasonal trends and property crimes in Minneapolis, 1987–1988. A moderator-variable time-series analysis of routine activities. J. Environ. Psychol..

[6] Baron R A, Bell P A (1976). Aggression and heat: the influence of ambient temperature, negative affect, and a cooling drink on physical aggression. J. Personality Soc. Psychol..

[7] Nguyen P-Y, Astell-Burt T, Rahimi-Ardabili H, Feng X (2021). Green space quality and health: a systematic review. Int. J. Environ. Res. Public Health.

[8] Taylor L, Hochuli D F (2017). Defining greenspace: multiple uses across multiple disciplines. Landsc. Urban Plan..

[9] Bogar S, Beyer K M (2016). Green space, violence, and crime: a systematic review. Trauma Violence Abuse.

[10] Venter Z S, Shackleton C, Faull A, Lancaster L, Breetzke G, Edelstein I (2022). Is green space associated with reduced crime? A national-scale study from the Global South. Sci. Total Environ..

[11] Akers T A, Potter R H, Potter R H, Hill C V (2012). Epidemiological Criminology: A Public Health Approach to Crime and Violence.

[12] Shepley M, Sachs N, Sadatsafavi H, Fournier C, Peditto K (2019). The impact of green space on violent crime in urban environments: an evidence synthesis. Int. J. Environ. Res. Public Health.

[13] Lee S, Koo B W, Kim Y (2023). Associations between tree characteristics and street crime: a case study in downtown Austin, TX. Urban For. Urban Green.

[14] Mak B K L, Jim C Y (2018). Examining fear-evoking factors in urban parks in Hong Kong. Landsc. Urban Plan..

[15] Huang W, De Roos A J, Kondo M C, Clougherty J E, Zhao Y, Schinasi L H (2024). Gender and violent crime modify associations between greenspace and cardiovascular disease mortality in Philadelphia PA. Health Place.

[16] Kondo M C, Clougherty J E, Hohl B C, Branas C C (2021). Gender differences in impacts of place-based neighborhood greening interventions on fear of violence based on a cluster-randomized controlled trial. J. Urban Health.

[17] Lin J, Wang Q, Huang B (2021). Street trees and crime: what characteristics of trees and streetscapes matter. Urban For. Urban Green.

[18] Wang R, Cleland C L, Weir R, McManus S, Martire A, Grekousis G, Bryan D, Hunter R F (2024). Rethinking the association between green space and crime using spatial quantile regression modelling: do vegetation type, crime type, and crime rates matter?. Urban For. Urban Green.

[19] Wu Z, Chen L (2017). Optimizing the spatial arrangement of trees in residential neighborhoods for better cooling effects: integrating modeling with *in-situ* measurements. Landscape Urban Plan..

[20] Wong N H, Tan C L, Kolokotsa D D, Takebayashi H (2021). Greenery as a mitigation and adaptation strategy to urban heat. Nat. Rev. Earth Environ..

[21] US Census Bureau (2020). Census Redistricting Data 2020. https://www.census.gov.

[22] Schnell C, Braga A A, Piza E L (2017). The influence of community areas, neighborhood clusters, and street segments on the spatial variability of violent crime in Chicago. J. Quant. Criminol..

[23] Cruz E, D’Alessio S J, Stolzenberg L (2023). The effect of maximum daily temperature on outdoor violence. Crime Delinquency.

[24] Chicago Police Department (2025). Crimes - 2001 to Present. Chicago Data Portal.

[25] PRISM Climate Group (2022). PRISM climate data. https://www.prism.oregonstate.edu/.

[26] MRLC (2024). Multi-resolution land characteristics consortium—national land cover database (NLCD). https://www.mrlc.gov/data/nlcd-land-cover-conus-all-years.

[27] OpenStreetMap (2023). United States of America geofabrik.

[28] Zhou Q, Wang S, Liu Y (2022). Exploring the accuracy and completeness patterns of global land-cover/land-use data in OpenStreetMap. Appl. Geogr..

[29] Gelmi‐Candusso T A, Rodriguez P, Fidino M, Rivera K, Lehrer E W, Magle S, Fortin M-J (2024). Leveraging open‐source geographic databases to enhance the representation of landscape heterogeneity in ecological models. Ecol. Evol..

[30] Arsanjani J J, Vaz E (2015). An assessment of a collaborative mapping approach for exploring land use patterns for several European metropolises. Int. J. Appl. Earth Observ. Geoinf..

[31] Klompmaker J O (2024). Associations of street-view greenspace with Parkinson’s disease hospitalizations in an open cohort of elderly US medicare beneficiaries. Environ. Int..

[32] Kondo M C, South E C, Branas C C, Richmond T S, Wiebe D J (2017). The association between urban tree cover and gun assault: a case-control and case-crossover study. Am. J. Epidemiol..

[33] U.S. Census Bureau (2025). American Community Survey 5-year Data (2011-2022) U.S. Census Bureau.

[34] Heo S, Afanasyeva Y, Liu M, Mehta-Lee S, Yang W, Trasande L, Bell M L, Ghassabian A (2024). Prenatal exposure to residential greenness, fetal growth, and birth outcomes: a cohort study in New York City. Am. J. Epidemiol..

[35] Holzer K J, AbiNader M A, Vaughn M G, Salas-Wright C P, Oh S (2022). Crime and violence in older adults: findings from the 2002–2017 national survey on drug use and health. J. Interpersonal Violence.

[36] Mitchell R, Popham F (2008). Effect of exposure to natural environment on health inequalities: an observational population study. Lancet.

[37] Gasparrini A, Armstrong B, Kenward M G (2012). Multivariate meta‐analysis for non‐linear and other multi‐parameter associations. Stat. Med..

[38] Gasparrini A, Armstrong B (2010). Time series analysis on the health effects of temperature: advancements and limitations. Environ. Res..

[39] Cao J, Valois M-F, Goldberg M S (2006). An S-plus function to calculate relative risks and adjusted means for regression models using natural splines. Comput. Methods Prog. Biomed..

[40] Lyons V H, Gause E L, Spangler K R, Wellenius G A, Jay J (2022). Analysis of daily ambient temperature and firearm violence in 100 US cities. JAMA Network Open.

[41] Hou K, Zhang L, Xu X, Yang F, Chen B, Hu W, Shu R (2023). High ambient temperatures are associated with urban crime risk in Chicago. Sci. Total Environ..

[42] DeLisi M, Alcala J, Kusow A, Hochstetler A, Heirigs M, Caudill J, Trulson C, Baglivio M (2017). Adverse childhood experiences, commitment offense, and race/ethnicity: are the effects crime-, race-, and ethnicity-specific?. Int. J. Environ. Res. Public Health.

[43] Zheng S, He C, Guldmann J M, Xu H, Liu X (2023). Heat mitigation benefits of urban trees: a review of mechanisms, modeling, validation and simulation. Forests.

[44] Taleghani M (2018). Outdoor thermal comfort by different heat mitigation strategies- A review. Renew. Sustain. Energy Rev..

[45] Santamouris M, Ding L, Fiorito F, Oldfield P, Osmond P, Paolini R, Prasad D, Synnefa A (2017). Passive and active cooling for the outdoor built environment—analysis and assessment of the cooling potential of mitigation technologies using performance data from 220 large scale projects. Sol. Energy.

[46] Jia S, Wang Y (2021). Effect of heat mitigation strategies on thermal environment, thermal comfort, and walkability: a case study in Hong Kong. Build. Environ..

[47] Salmond J A (2016). Health and climate related ecosystem services provided by street trees in the urban environment. Environ. Health.

[48] Wu Q, Huang Y, Irga P, Kumar P, Li W, Wei W, Shon H K, Lei C, Zhou J L (2024). Synergistic control of urban heat island and urban pollution island effects using green infrastructure. J. Environ. Manage..

[49] Markevych I (2017). Exploring pathways linking greenspace to health: theoretical and methodological guidance. Environ. Res..

[50] Smiley K T, Sharma T, Steinberg A, Hodges-Copple S, Jacobson E, Matveeva L (2016). More inclusive parks planning: park quality and preferences for park access and amenities. Environ. Justice.

[51] Potgieter A, Fabris-Rotelli I N, Breetzke G, Wright C Y (2022). The association between weather and crime in a township setting in South Africa. Int. J. Biometeorol..

[52] Manickathan L, Defraeye T, Allegrini J, Derome D, Carmeliet J (2018). Parametric study of the influence of environmental factors and tree properties on the transpirative cooling effect of trees. Agric. For. Meteorol..

[53] Ju Y, Dronova I, Ma Q, Lin J, Moran M R, Gouveia N, Hu H, Yin H, Shang H (2024). Assessing normalized difference vegetation index as a proxy of urban greenspace exposure. Urban For. Urban Green.

[54] Rahman M A, Hartmann C, Moser-Reischl A, Von Strachwitz M F, Paeth H, Pretzsch H, Pauleit S, Rötzer T (2020). Tree cooling effects and human thermal comfort under contrasting species and sites. Agric. For. Meteorol..

